# Low-risk trials for children and pregnant women threatened by unnecessary strict regulations. Does the coming EU Clinical Trial Regulation offer a solution?

**DOI:** 10.1007/s00431-020-03715-3

**Published:** 2020-06-13

**Authors:** Max Knaapen, Martine Corrette Ploem, Maya Kruijt, Martijn A. Oudijk, Rieke van der Graaf, Pierre M. Bet, Roel Bakx, L. W. Ernst van Heurn, Ramon R. Gorter, Johanna H. van der Lee

**Affiliations:** 1 Department of Paediatric Surgery, Emma Children’s Hospital, Amsterdam UMC, University of Amsterdam & Vrije Universiteit Amsterdam, Amsterdam, The Netherlands; 2 Department of Social Medicine, Section Health Law, Amsterdam UMC, University of Amsterdam, Amsterdam, The Netherlands; 3 Department of Reproductive Medicine, Amsterdam Reproduction and Development Research Institute, Amsterdam UMC, University of Amsterdam and Stichting Zorgevaluatie Nederland, Amsterdam, The Netherlands; 4 Department of Obstetrics, Amsterdam Reproduction and Development Research Institute, Amsterdam UMC, University of Amsterdam and Dutch Consortium for Healthcare Evaluation and Research in Obstetrics and Gynaecology, Amsterdam, The Netherlands; 5 Department of Medical Humanities, Julius Center for Health Sciences and Primary Care, University Medical Center Utrecht, Utrecht, The Netherlands; 6 Department of Clinical Pharmacology and Pharmacy, VU University Medical Centre, Amsterdam UMC, Vrije Universiteit, Amsterdam, The Netherlands; 7 Paediatric Clinical Research Office, Emma Children’s Hospital, Amsterdam UMC, University of Amsterdam, Amsterdam, The Netherlands; 8 Knowledge Institute of the Dutch Association of Medical Specialists, Utrecht, The Netherlands

**Keywords:** Clinical trial regulation, Pragmatic clinical trials, Investigator initiated research, ICH-GCP guideline, Risk-based trial regulation

## Abstract

Investigator-initiated clinical trials are crucial for improving quality of care for children and pregnant women as they are often excluded from industry-initiated trials. However, trials have become increasingly time-consuming and costly since the EU Clinical Trial Directive entered into force in 2001. This directive made compliance with ICH-Good Clinical Practice Guidelines (ethical and quality standard for conducting human subject research) mandatory for all clinical trials, regardless of its risk-classification. By discussing two investigator-initiated, ‘low-risk’ drug trials, we aim to illustrate that compliance with all GCP requirements makes trials very laborious and expensive, while a clear rationale is missing. This discourages clinical researchers to start and carry out investigator-initiated research. However, the forthcoming EU Clinical Trial Regulation (No 536/2014) seems to provide a solution as it allows for less stringent rules for low-risk trials. We want to raise awareness for these developments in both the clinical research community and the European and national regulatory authorities. Implementation of this forthcoming Regulation regulatory policies should be done in such a way that investigator-initiated trials evaluating standard care interventions will become more feasible. This will allow us to obtain evidence on optimal and safe treatments, especially for groups that are underrepresented in medical research.

**Table:** 

**What is Known** • *Investigator-initiated trials are indispensable for improving care for children and pregnant women as they are often excluded from industry-initiated trials* • *Trials have become increasingly time-consuming and costly because of mandatory compliance with ICH-GCP guidelines*	
**What is New** • *The forthcoming EU Clinical Trial Regulation allows less stringent rules for low-risk trials* • *The national legislator and regulatory authorities should recognize the importance of this opportunity and implement the Regulation in such a way that investigator-initiated trials will become more feasible*

**What is Known**

• *Investigator-initiated trials are indispensable for improving care for children and pregnant women as they are often excluded from industry-initiated trials*

• *Trials have become increasingly time-consuming and costly because of mandatory compliance with ICH-GCP guidelines*

**What is New**

• *The forthcoming EU Clinical Trial Regulation allows less stringent rules for low-risk trials*

• *The national legislator and regulatory authorities should recognize the importance of this opportunity and implement the Regulation in such a way that investigator-initiated trials will become more feasible*

## Introduction

Evidence-based medicine has proven instrumental in improving healthcare, with the randomized clinical trial (RCT) regarded as the highest level of evidence. Since the introduction of the EU Clinical Trials Directive in 2001 [1], trials are subject to a regulatory system aimed at transparency and accountability, incorporated in the ‘International Council for Harmonization Guideline for Good Clinical Practice’ (ICH-GCP). Since then, the performance of clinical trials has become increasingly complex, time-consuming and expensive [2, 3]. The largest independent cancer research network in Europe (EORTC) reported that after the directive’s introduction, trial costs increased by 85% and the number of new trials fell by 63% [4]. Data from EU research institutes show a similar effect, as the percentage of investigator-initiated trials declined from 40 to 14% [5]. As a result, there is an increasing interest in nonrandomized (observational) studies to circumvent the administrative and financial hurdles that come with RCTs, though at the risk of biased results [6].

Commercial parties, such as pharmaceutical companies, are less inclined to invest in expensive research with little commercial value [7], such as research on children, pregnant women and other vulnerable groups. The latter results in a relative underrepresentation of these groups in clinical trials [8, 9]. To improve the quality of care and reduce healthcare expenses for these groups, academic researchers, working in a non-profit sector, need to perform their own studies [10]. However, the ‘administrative burden’ resulting from regulations and oversight procedures is becoming a critical obstacle in investigator-initiated research.

The principles that are at the core of our legislation for human subject research, such as that the health of my patient is my first consideration and that research must be conducted by qualified professionals, are beyond reproach. However, demanding full compliance with the ICH-GCP guidelines in studies with only minimal risks does not seem justified. By demonstrating some of the consequence experienced in current research practice, we hope to raise awareness of the effects the ensuing administrative burden has on research [11]. In this context, we will discuss the forthcoming EU Clinical Trial Regulation (No 536/2014) [12]. This new regulation has been adopted and entered into force in 2014, but has yet to come into application. This application will happen 6 months after the European Commission has published notice of a successful internal audit of a new EU portal that will ‘streamline and facilitate the flow of information between sponsors and Member States’. This audit is set to commence in December 2020 [13]. When the regulation becomes applicable, it will replace the current 2001 Clinical Trial Directive and the national legislation that was put in place to implement the 2001 Directive. Therefore, EU member countries and national regulatory authorities are now preparing for the introduction of the new Clinical Trial Regulation (CTR) in their countries. This article addresses and illustrates how the current legislation and the ensuing administrative burden disproportionally affects research practice and threatens the generation of necessary evidence in vulnerable populations [11]. Secondly, we discuss how the more ‘risk-based’ regulatory approach set out in the new EU CTR might offer a solution.

## Limitations of the ‘one-size-fits-all’ regulatory framework

The ICH-GCP guidelines—on which the current oversight regulations are based—were predominantly developed to oversee high-risk commercial placebo-controlled trials evaluating new medical products pursuing market authorization [3, 14]. Unfortunately, these guidelines do not take into account the fact that the clinical trials they regulate can substantially differ in the extent to which they pose physical and/or mental risks to participants. The practical consequences of these regulations are especially evident in so called ‘pragmatic trials’—evaluating well-known, clinical strategies generally applied in present-day healthcare [15]. This type of trial generally causes no or very little additional risks for their participants, but still needs to comply with the same detailed rules as trials with new pharmaceutical compounds with unknown safety profiles.

To illustrate the far-reaching implications of the current regulatory system for research practice, two examples of pragmatic multicentre RCTs are presented here. Both are publicly funded and designed, initiated and carried out by academic researchers. The APAC trial compares initial antibiotic treatment to immediate appendectomy in children [16]. APOSTEL 8 is a placebo-controlled trial for the treatment of threatened preterm birth with the registered tocolytic drug, atosiban, which has been used for this indication since 2000.

### Mandatory adherence to ICH-GCP and Good Manufacturing Practice guidelines

According to the 2001 clinical trial directive, any investigation in human subjects to verify the clinical, pharmacological and/or other pharmacodynamic effects of an investigational product is considered a clinical trial and should thus comply with the ICH-GCP guidelines, including Good Manufacturing Practice (GMP) [1]. This requirement has substantial consequences for the APAC trial, as the administered antibiotic needs to be considered as an investigational medical product (IMP), regardless of the fact that this concerns an open-label, low-risk trial, with amoxicillin/clavulanic-acid used within its licenced indication and dosage. These requirements are listed in the GMP’s Annex 13 [17], a few of its principles are summarized in Text box 1. An overview of the ensuing consequences, most of which are in place to ensure drug traceability, is provided in Table 1.

**Table 1:** Practical consequences of the GMP regulation for the use of an IMP in a clinical trial

1. Requirements for product labelling	- Drafting a Product Specification File - Drafting a production/labelling protocol - Acquisition of the medication (instead of reimbursement by insurance companies when prescribed for regular healthcare) - Production (labelling) of the IMP - Certification of the IMP batch by a qualified person *All actions above need to be repeated if the IMP expires during the course of the trial.
2. Additional requirements participating site	- Pharmacy agreements - Temperature controlled distribution of IMP by GCP-accredited transportation company - Separate and conditioned storage in local pharmacies - Acquisition of dedicated temperature loggers for IMP storage on the ward or emergency room (to allow for access outside pharmacy opening hours) - Temperature logs and procedures for detecting and dealing with temperature variations, like quarantine medication or destruction. - Audit of ward storage of the IMP
3. Added responsibilities healthcare professionals	- On paper documentation of each administered dose, including batch number, dosage, expiration date, including a signature - All nurses administering IMP need to be GCP trained and delegated, including signatures of principal investigator - Separate prescription routines for usage of the IMP - Pharmacy documentation on drug accountability

The rational for many of the GMP regulations seems difficult to discern when it concerns medical products that have already been licenced for a considerable time, which means that, next to clinical experience, extensive information is available on its stability and production process. This applies in particular to trials where the use of medication can be considered standard care and risks for participants are to a very large extent predictable. Complying with all the regulations makes the conduct of these trials unnecessarily laborious and complex and therefore needlessly expensive. Usage of amoxicillin/clavulanic-acid as an IMP costs at least 268 euros per patient, including costs for local pharmacies, labelling and distribution. Using regular high-volume stock medication would cost at least seven times less (37 euros per patient). In the APAC trial, the IMP-related costs account for 23% of the total trial budget; for the APOSTEL 8, this even exceeds 50% of the trial budget.

### Mandatory certified GCP training for healthcare professionals performing any study-related task

The obligation to have adequately trained study staff is set out in the EU GCP directive (2005/28/EC) [18], stating: ‘Each individual involved in conducting a trial shall be qualified by education, training, and experience to perform his tasks’. If this is interpreted stringently, as is done by for instance the Dutch health inspectorate and the UK Medicines and Healthcare products Regulatory Agency, any health professional involved in the informed consent process, administering an IMP or recording study data, needs to have documented and up-to-date knowledge on all research-related legislation and ICH-GCP guidelines including, for instance, knowledge on import licences for IMPs or the route to apply for a CE marking for medical devices.

The issue of this mandatory training is well illustrated in the APOSTEL 8, and—in fact—in all studies on threatened preterm birth or other trials that include patients in the (semi) acute setting. As these patients usually present in the hospital outside regular working hours, many of the study-related tasks must be performed by the ‘on-call’ team. As a result, all members of all labour ward teams need to have completed both study-specific training and training on legislation and ICH-GCP guidelines. APOSTEL 8 is an international trial with over 40 participating sites. This implies that hundreds of doctors, midwives and research nurses in many different locations need to be trained and certified on subjects that have no relation to their study task. Additionally, each healthcare professional needs to be registered on a site signature delegation log, including their signature and a signature from the local principal investigator. These obligations are practically impossible to comply with in a (semi) acute setting, unless there is a dedicated and fully trained study team on standby around the clock to perform all study tasks.

## Towards ‘risk-based’ regulations

Hopefully, the above illustrates how the ‘one-size-fits-all’ approach of the current regulatory system makes the performance of low-risk research very time-consuming and costly without serving its primary goal: ensuring medical progress by carrying out research, while protecting research subjects against risks and burden. Therefore, the medical community has—more than once—called for a more proportionate and appropriate set of rules warranting accountability and transparency [3, 4, 14, 19, 20]. The forthcoming 2014 EU CTR might offer a solution as it introduces a new ‘low-intervention clinical trial’ regulatory framework [12]. It states (recital 11): ‘*[m] any clinical trials pose only a minimal additional risk to subject safety compared to normal clinical practice. Particularly when the IMP is covered by a marketing authorization [ …] Those low-intervention clinical trials are often of crucial importance for assessing standard treatments and diagnoses […] contributing to a high level of public health. Those clinical trials should be subject to less stringent rules, as regards monitoring, requirements for the contents of the master file and traceability of IMPs’.* The areas that will be positively affected by the new regulation and the probable practical consequences are summarized in Table 2, based on the regulation [12], question and answer draft [21] and the expert group recommendation [22]. All the conditions to classify as a ‘low-interventional clinical trial’ and thus will be subject to less stringent rules are stated in the CTR. article 2. par 3 and are the following:

- *the investigational medicinal products, excluding placebos, are authorised;*
- *according to the protocol, (i) the IMPs are used in accordance with the marketing authorisation; or (ii) the use of the IMPs is evidence-based and supported by published scientific evidence on the safety and efficacy of those IMPs in any of the Member States concerned; and*
- *the additional diagnostic or monitoring procedures do not pose more than minimal additional risk or burden to the safety of the subject compared to normal clinical practice in any member state concerned;*

**Table 2:** Potential practical consequences of the EU Clinical Trial Regulation (No 536/2014) [12] on trial conduct, based on the regulation [12], question and answer draft [21] and the expert group recommendation [22]

Impacted area (CTR mentioning)	Practical consequence
Safety reporting (Article 41.2, Annex III 2.5, 21)	Adverse events that can expected with the intervention, disease or population may be waived from recording if justified in the protocol and supported by the risk assessment outcome. Meaning only certain adverse events need to be recorded and reported. This applies in particular to marketed IMPs, dependent on how much is known on its usage in a certain population or disease.
IMP management (Article 51.2)	The sponsor can decide that normal prescribing practice and documentation can suffice. Prescribed amounts and doses taken may be taken from a medical chart or other documents, e.g. the patient’s diary, case record form or the routine pharmacy documentation. In case of blinded clinical trials, sufficient traceability and documentation should be available to allow for a recall. Other risk factors, like the stability of the active ingredient should also be considered in the risk assessment and for example, temperature monitoring or light-protection.
IMP labelling (Article 57)	No additional labelling should be required for clinical trials that do not involve the blinding of the label.
Trial monitoring (Article 48)	The sponsor should adequately monitor the conduct of a clinical trial. The extent and nature of the monitoring shall be determined by the sponsor on the basis of an assessment that takes into consideration all characteristics of the clinical trial, including; a. whether it is a low-intervention clinical trial; b. the objective and methodology of trial; c. the degree of deviation of the intervention from normal clinical practice.
Trial documentation (Article 57)	The clinical trial master file shall contain essential documents which allow verification of the conduct of a clinical trial and the quality of the data generated, taking into account whether the clinical trial is low-interventional. Documents can be omitted that are no longer necessary following, for example, less extensive IMP management and monitoring.

Sponsor—initiator of the study

The exact definition of low-risk trials that can be subjected to this less stringent regulatory regime will determine to what extent the new CTR will bring about less administrative burden. This will depend on how national regulatory authorities interpret the conditions set out in the CTR article 2. par 3. The most important decision will be whether a treatment is considered to be ‘supported by published scientific evidence’ and to what extent additional risks and burden will be regarded as ‘minimal compared to normal clinical practice’. This could be a threat to paediatric and obstetric research as many medical products have not been specifically tested in children or pregnant women and many risk classification tools consider research with these populations to be high risk by default. If this strict interpretation is maintained, it is unlikely that low-risk paediatric and obstetric research can benefit from the less stringent regulatory regime laid out in the CTR.

As for the condition ‘minimal additional risks or burden’, some guidance is provided by the EU commission’s expert group on clinical trials [22]. They specify the following examples of diagnostic/monitoring procedures that can be considered as a minimal additional burden: ‘weighing, height measuring, questionnaires, analysis of saliva, urine, stool samples, EEG and ECG measurements, blood withdrawal through a pre-existent catheter or with minimal additional venepuncture’. Unfortunately, the expert group recommendations are accompanied by a warning that the document does not necessarily reflect the views of the European Commission and should not be interpreted as a commitment to any official initiative. Therefore, it remains unclear to what extent they can be regarded as an official explanation of the terminology of article 2. How this condition is eventually implemented will again depend on the interpretation by the national regulatory authorities. Strict interpretation of the conditions for low-risk clinical trials would mean that the generation of essential evidence for the treatment of vulnerable populations is made almost impossible.

### Similar ‘risk-based’ approach towards training requirements and GCP certification is missing

There is a clear rationale why clinical researchers need to have verifiable knowledge of ICH-GCP guidelines and legislation. However, the current regulations mandate healthcare professionals, performing even the smallest study task, to have verifiable training in all ICH-GCP guideline–related topics. Would it not be more appropriate that this requirement is tailored to the professional’s specific role and study specific activities? Apart from the costs and administration involved with certification, regulations such as these do not seem compatible with extensive teams that perform limited study tasks alongside their regular clinical work, especially in the (semi) acute setting.

Unfortunately, the CTR fails to address this issue directly. Article 49 states that individuals involved in conducting a clinical trial should be *suitably* qualified by education, training and experience to perform their tasks [12]. This seems to leave room for a proportionate approach; however, a proportionate approach for low-interventional trials is not mentioned explicitly. A more ‘task-tailored’ approach in training requirements to commensurate with the roles and responsibilities of the study staff would be optimal. This recommendation is in line with a joint statement released by the UK Medicines and Healthcare products Regulatory Agency & Health Research Authority, which acknowledges that researchers are disproportionately and inappropriately burdened with the consequences of having to comply with ICH-GCP guidelines [23]. We urge the regulatory authorities to set similar standards for a ‘task-tailored’ approach, as opposed to the current policies that demand extensive training and certification regardless of the—sometimes very limited—study tasks healthcare professionals perform.

## In conclusion

We hope that our experiences with the two trials presented in this paper offer compelling evidence that it is time for a paradigm shift. Such a shift implies that the current one-size-fits-all approach, which dominates current clinical trial oversight, is replaced by a risk-based approach. We expect that this could give a substantial impulse to low-risk investigator-initiated trials, which are currently more and more discouraged by unnecessarily time-consuming and expensive ICH-GCP guideline recruitments. These trials are indispensable for obtaining evidence on optimal and safe treatments for patients, specifically groups that are underrepresented in current medical research, such as children and pregnant women. We hope that both the clinical research community and the European and national regulatory authorities see the urgency of this problem and provide clarity about the room for less stringent monitoring and safety regulations for low-risk trials, as provided in the forthcoming EU Clinical Trial Regulation. We urge regulatory authorities to handle these conditions, needed to classify as low-risk, in such a way that it does not excluded research with children and pregnant women. We also ask for a ‘task-tailored’ approach towards (GCP) training requirements as the current one-size-fits-all approach. The current requirements makes research with extensive teams that perform only limited study tasks alongside their regular clinical work almost impossible. This will allow us to obtain evidence on optimal and safe treatments, especially for groups that are underrepresented in medical research.

## Abbreviations

CTR: Clinical Trial Regulation
GMP: Good manufacturing practice
ICH-GCP: International Council for Harmonization Guideline for Good Clinical Practice
IMP: Investigational medical product
RCT: Randomized controlled trials
